# Exploring the evolutionary dynamics of plasmids: the *Acinetobacter *pan-plasmidome

**DOI:** 10.1186/1471-2148-10-59

**Published:** 2010-02-24

**Authors:** Marco Fondi, Giovanni Bacci, Matteo Brilli, Maria Cristiana Papaleo, Alessio Mengoni, Mario Vaneechoutte, Lenie Dijkshoorn, Renato Fani

**Affiliations:** 1 Laboratory of Microbial and Molecular Evolution, Dept. of Evolutionary Biology, Via Romana 17-19, University of Florence, I-50125 Florence, Italy; 2 Laboratoire de Biometrie et Biologie Evolutive, UMR CNRS 5558, Lyon, France; 3 Laboratory Bacteriology Research, Faculty Medicine & Health Sciences, University of Ghent, Belgium; 4 Department of Infectious Diseases, Leiden University Medical Center, PO Box 9600, 2300 RC, Leiden, The Netherlands

## Abstract

**Background:**

Prokaryotic plasmids have a dual importance in the microbial world: first they have a great impact on the metabolic functions of the host cell, providing additional traits that can be accumulated in the cell without altering the gene content of the bacterial chromosome. Additionally and/or alternatively, from a genome perspective, plasmids can provide a basis for genomic rearrangements *via *homologous recombination and so they can facilitate the loss or acquisition of genes during these events, which eventually may lead to horizontal gene transfer (HGT). Given their importance for conferring adaptive traits to the host organisms, the interest in plasmid sequencing is growing and now many complete plasmid sequences are available online.

**Results:**

By using the newly developed Blast2Network bioinformatic tool, a comparative analysis was performed on the plasmid and chromosome sequence data available for bacteria belonging to the genus *Acinetobacter*, an ubiquitous and clinically important group of γ-proteobacteria. Data obtained showed that, although most of the plasmids lack mobilization and transfer functions, they have probably a long history of rearrangements with other plasmids and with chromosomes. Indeed, traces of transfers between different species can be disclosed.

**Conclusions:**

We show that, by combining plasmid and chromosome similarity, identity based, network analysis, an evolutionary scenario can be described even for highly mobile genetic elements that lack extensively shared genes. In particular we found that transposases and selective pressure for mercury resistance seem to have played a pivotal role in plasmid evolution in *Acinetobacter *genomes sequenced so far.

## Background

Plasmids are among the most important players in the evolution of prokaryotes and in their adaptation to fluctuating environmental conditions [1-3]. They are actually involved in many accessory functions and constitute, together with "not essential" chromosomal regions, what is referred to as the "dispensable genome" in the microbial pan-genome concept [4]. Typically, a plasmid includes one or more essential genes encoding replicative functions. In addition, it may harbor one or more genes coding for a variable panoply of accessory metabolic processes and functions that are, in general, different from those encoded by chromosome(s) [2,5,6]. Actually, plasmid architecture is more flexible than the chromosomal one, concerning both gene content and gene organization, even within members of the same bacterial genus. Plasmids genes are in fact under differential selection while moving through the prokaryotic community [3], and consequently, they frequently gain and lose genes, revealing a very dynamic organization [2,7,8]. This flexibility is mostly due to the abundance of transposable elements they harbor and that facilitate intra- and intermolecular recombination by creating homology regions. Moreover, plasmids can be both vertically and horizontally inherited in a prokaryotic community, giving rise to the possibility that the very same plasmid molecule can be hosted in different genomic contexts, boosting the rearrangement of their functions and of gene organization [9-11].

Despite the key-role of plasmids in the prokaryotic world, the evolutionary dynamics of plasmids have been poorly explored, mainly because of the lack of extensive similarities between them, except for genes involved in replication and transfer functions [12,13], which hampers classical phylogenetic analyses based on gene genealogy and synteny [14]. However, a computational biology approach (Blast2Network) based on similarity networks reconstruction and phylogenetic profiling has been recently proposed and applied in a study-case to depict the similarities among plasmids from *Enterobacteriaceae *[15]. The bioinformatic package Blast2Network (hereafter designated B2N) provides an immediate visualization of the similarities, existing among aminoacidic or nucleic sequences [15]. This, in turn, opens the possibility to trace the evolutionary dynamics and history of entire plasmids and not only of single genes and/or operons harbored by them. In this context, bacteria belonging to the genus *Acinetobacter *may represent an excellent study-case, because strains of this genus are commonly found in soil, water and in association with animals [16,17]. Besides, some of them are well-known human pathogens, often responsible for opportunistic infections in hospitalized patients [16,18,19]. A striking recent manifestation of *A. baumannii *is the occurrence in severely wounded soldiers coming back from Iraq [20]. Currently, the genus *Acinetobacter *comprises 19 species with valid names and at least 13 putative species [21]. More than 975 strains have been recorded in the Taxonomy Browser of NCBI at July, 2 2009, but the precise taxonomy of these strains is not always clear since many have not been identified by unambiguous genotypic identification methods [21,22]. *Acinetobacter *strains are of special interest for the huge variety of environments they can colonize and the diverse metabolic abilities they display, as inferred from the occurrence of, e.g., hydrocarbon degrading strains in oil spills, human pathogens resistant to a plethora of antibiotics, rhizospheric bacteria and strains inhabiting bioreactors or insect guts [6,23-29]. Moreover, a special interest for members of this genus also relies on the ability of some strains, i.e. those belonging to the species *A. baylyi*, to undergo natural transformation [9,30]. This attribute has made the *A. baylyi *strain ADP1 (also named BD413) an exceptional tool for genetic analysis and engineering [31].

It has been reported that several *Acinetobacter *strains, especially those sharing particular ecological niches that require specific adaptations, like polluted environments and bioreactors, harbor plasmid molecules of different sizes undergoing frequent molecular rearrangements [32-34]. Particularly interesting among *Acinetobacter *plasmids is the pKLH2 family [35], a group of evolutionary related plasmids harboring mercury resistance genes (*mer*) embedded in a single compact operon that, in turn, has been suggested to represent an aberrant mercury resistance transposon (namely TndPKHLK2) that has lost genes responsible for transposition [36]. Recently, some *Acinetobacter *genomes and plasmids have been completely sequenced. On March 31, 2009, the sequences of 7 genomes and 29 plasmids were available (Table 1). The *Acinetobacter *"pan-plasmidome", that is the complete set of plasmids harbored by members of this genus (comprising plasmids isolated from both pathogenic and environmental strains), is then particularly attractive to study its evolutionary dynamics because of the eclectic lifestyle of their host strains and the possible frequent genetic exchanges between its members.

**Table 1:** List of completely sequenced *Acinetobacter *plasmids and chromosomes used in this work.

Strains		Plasmids					Chromosomes			
Species and/or designation	Origin	n.	Name	A.N.	Length (bp)	ORF(s)	n.	Length (bp)	A.N.	ORF(s)
*Acinetobacter baumannii*	Clin.	1	pABIR	NC_010481	29823	26	n.d.			
*Acinetobacter baumannii *ATCC19606^T^	Clin.	1	pMAC	NC_006877	9540	11	n.d.			
* Acinetobacter baumannii*	Clin.	1	pAB02	AY228470	4162	6	n.d.			
*Acinetobacter baumannii *ACICU	Clin.	2	pACICU1	NC_010605	28279	28	1	3904116	NC_010611	3667
			pACICU2	NC_010606	64366	64				
*Acinetobacter baumannii *ATCC 17978	Clin.	2	pAB1	NC_009083	13408	11	1	3976747	NC_009085	3351
			pAB2	NC_009084	11302	5				
*Acinetobacter baumannii *AYE	Clin.	4	p1ABAYE	NC_010401	5644	7	1	3936291	NC_010410	3607
			p2ABAYE	NC_010402	9661	11				
			p3ABAYE	NC_010403	94413	82				
			p4ABAYE	NC_010404	2726	5				
*Acinetobacter baumannii *SDF	Body lice	3	p1ABSDF	NC_010395	6106	8	1	3421954	NC_010400	2913
			p2ABSDF	NC_010396	25014	30				
			p3ABSDF	NC_010398	24922	24				
*Acinetobacter baumannii *AB0057	Clin.	1	pAB0057	NC_011585	8729	11	1	4050513	NC_010410	3790
*Acinetobacter baumannii *AB307-0294	Clin.	0					1	3760981	NC_011595	3451
*Acinetobacter *sp. EB104	Unknown	1	pAC450	NC_002760	4379	4	n.d.			
*Acinetobacter *sp. SUN	Clin.	1	pRAY	NC_000923	6076	10	n.d.			
*Acinetobacter venetianus*	Env.	2	pAV1	NC_010309	10820	11	n.d.			
			pAV2	NC_010310	15135	16				
*Acinetobacter sp *LUH5605	Env.	1	Ptet5605	AY228470	3727	4	n.d.			
*Acinetobacter *sp BW3	Env.	1	pKLH207	AJ486856	9910	16	n.d.			
*Acinetobacter calcoaceticus *KHW14	Env.	1	pKLH201	AJ251307	11191	14	n.d.			
*Acinetobacter calcoaceticus *KHP18	Env.	1	pKLH2	AF213017	6838	12	n.d.			
*Acinetobacter *sp ED23-35	Env.	1	pKLH208	AJ251272	9435	15	n.d.			
*Acinetobacter *sp ED45-25	Env.	1	pKLH205	AJ459234	8561	13	n.d.			
*Acinetobacter junii*	Env.	1	pKLH203	AJ486855	7195	12	n.d.			
*Acinetobacter *sp LS56-7	Env.	1	pKLH204	AJ487050	9489	15	n.d.			
*Acinetobacter lwoffii*	Env.	1	pKLH202	AJ486857	9471	17	n.d.			
*Acinetobacter *sp YAA*	Env.	1	pYA1	D86080	7407	5	n.d.			
*Acinetobacter baylyi *ADP1	Env.	0					1	3598621	NC_005966	3307
*Tot*.		29				493	7			24086

Sequences were downloaded from the NCBI web-site http://www.ncbi.nlm.nih.gov/genomes/genlist.cgi?taxid=2&type=1&name=Bacteria%20Complete%20Chromosomes and from the FTP repository ftp://ftp.ncbi.nih.gov/refseq/release/plasmid/ (as on 31st March 2009 ). N.d. "not determined", A.N. "accession number".

* These plasmids were retrieved in strains lacking a clear taxonomical affiliation [56,57]

Therefore, in this work, a detailed comparative analysis of the completely sequenced *Acinetobacter *plasmids, available in public databases, was performed with the aim to i) reconstruct their evolutionary dynamics and ii) investigate the evolutionary cross-talk between them and the chromosomes of *Acinetobacter *strains.

## Results

### Plasmid networks

**Figure 1:**
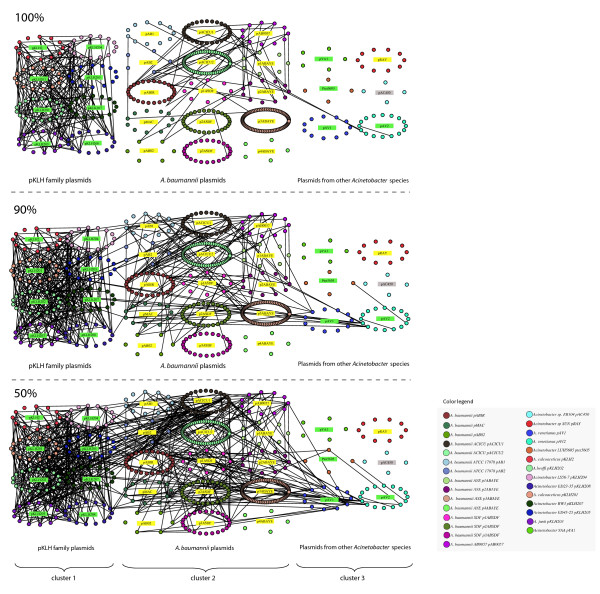
**Identity based networks of the 493 *Acinetobacter *plasmid encoded proteins**. All the proteins belonging to the same plasmid (nodes) are circularly arranged and are linked to the others according to their identity value. The resulting pictures for three different identity thresholds (100%, 90%, 50%) are shown. Plasmids names have been colored according to the habitat of their source microorganism: yellow indicates clinical sources, green indicates environmental sources, grey indicates that habitat information was not available.

The first aim of the work was the identification and the analysis of the possible evolutionary relationships existing among the 29 *Acinetobacter *plasmids. To this purpose, all the 493 retrieved sequences of *Acinetobacter *plasmid-encoded proteins were used as input for the B2N software (see Material and Methods), generating a set of networks showing all the sequence identities existing among these proteins. In these networks nodes represent proteins, whereas links indicate the existence of sequence identity among them (Figure 1 and [Additional file 1]). The degree of sequence identity threshold is *a priori *selected. In principle, the higher the threshold used, the lower the number of links existing between proteins encoded by different plasmids. In addition, it can be assumed that the higher the degree of aminoacid identity between two proteins, the more recent would be the event (recombination/transposition/duplication/vertical transmission) responsible for the presence of the two orthologous/paralogous coding genes in different plasmids. We selected a minimum of 50% identity threshold since this degree of sequence identity is sufficiently high to guarantee that in most cases the interconnected proteins perform the same function (i.e., they are encoded for by orthologous genes) [37,38]. The three networks shown in Figure 1 (at 100%, 90%, and 50% identity thresholds) and the other three reported in [Additional file 1]. (at 60%, 70%, and 80% identity thresholds) were obtained by reiterating the analysis using different identity thresholds.

#### Analysis of links

The analysis of the networks reported in Figure 1 revealed that:

a) as expected, the number of links and interconnected nodes decreased with the increase of identity threshold (Table 2). At 50% of sequence identity, 213 out of the 493 plasmid-encoded proteins were linked together. The other 280 proteins remained isolated because each of them did not share any link with the others and these were excluded from further analysis (see the Phylogenetic profiling section). The number of linked proteins decreased to 133 at a 100% sequence identity threshold. Still this number is unexpectedly high and suggests that the plasmids sharing at least one gene underwent recombination events very recently.

**Table 2:** Number of nodes and links at different identity threshold between 29 different *Acinetobacter *plasmids

Identity threshold (%)	Number of	
	Nodes	Links
50	213	534
60	201	501
70	193	471
80	187	462
90	174	384
100	133	228

b) Three main groups can be recognized (Figure 1): Cluster 1 includes the eight plasmids pKLH2, pKLH201, pKLH202, pKLH203, pKLH204, pKLH205, pKLH207, and pKLH208 (hereinafter designated as the pKLH-family plasmids) that are highly interconnected although they have been isolated from different *Acinetobacter *spp. strains (Table 1). Cluster 2 is constituted by fifteen plasmids isolated from 8 *A. baumannii *strains, and Cluster 3 includes the remaining six plasmids isolated from *Acinetobacter *species different from *A. baumannii*.

c) Four plasmids (pRAY, pAC450, pYA1, and p4ABAYE) harbor genes encoding proteins that do not share any link neither between them nor with other proteins in the network.

d) Concerning the connections within each group (intra-links), the analysis of Figure 1 reveals that plasmids of cluster 1 (pKLH family) maintain a very high number of links at the 100% threshold, whereas the number of intra-links of plasmids belonging to clusters 2 and 3 strongly decreases from the 50% to the 100% threshold. Overall, this finding suggests that pKLH plasmids share a more recent evolutionary pathway than that exhibited by the other plasmids. In addition to this, the finding that such pKLH plasmids have been isolated from different strains belonging to the same or to different *Acinetobacter *species suggests a high degree of horizontal flow of these plasmids (or at least of the shared genes). The biological significance of these data relies mainly on the fact that these plasmids harbor the genetic determinants for mercury resistance (*mer *genes, see [Additional file 2]) that are positively favored in an environment under a strong selective pressure, i.e. in the presence of high mercury concentrations.

e) Regarding the connections between plasmids belonging to different clusters (inter-links), no link was observed between pKLH-family plasmids and those belonging to the other two clusters at the 100% threshold. However, at the 90% and 50% thresholds, many inter-links between cluster 1 and cluster 2 plasmids were observed. This interconnection was mainly due to plasmid pACICU1 and involves proteins predicted to be involved in DNA transposition, recombination and replication. Interestingly proteins assigned to OXA-58 oxacillinase and AraC binding protein were shared between pACICU1 and pABIR up to the 90% identity threshold. The connections existing between cluster 2 and some cluster 3 plasmids were in some cases retained also at higher thresholds (90-100%), for instance with proteins of pAV1 and pAV2 plasmids from *A. venetianus*. These links at high threshold between clusters suggest that the plasmids involved shared at least some common steps in their evolutionary pathways.

f) In some cases, for instance plasmid pAV2, it is possible to recognize the traces of paralogous duplications within the same molecule.

g) The analysis of plasmids from the same strain revealed that, almost in all the cases, they did not share any link at the 100% threshold except for plasmids p2ABSDF and p3ABSDF (both from *A. baumannii *SDF), which had two links corresponding to two sequences assigned as "hypothetical proteins". The absence of links shared by these molecules may suggest the absence of recent genetic exchanges between plasmids in the same host.

#### Analysis of nodes

**Figure 2:**
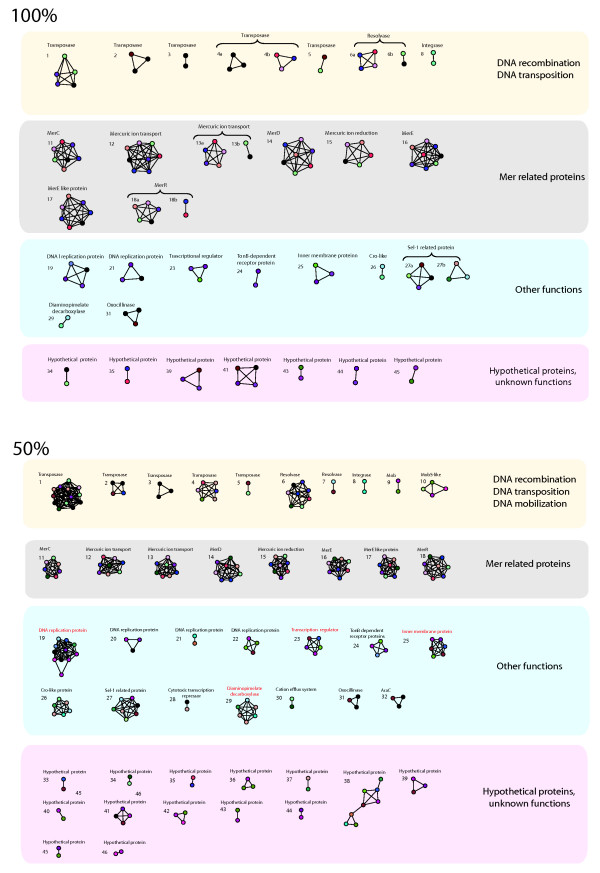
**Uniform visualization of the networks shown in Figure 1**. The different clusters embed proteins sharing 50% (below) and 100% (above) identity. Plasmids color legend as in Figure 1.

To analyze the functional classes of clustered/unclustered proteins, we made use of the uniform visualization output of the B2N software. The uniform visualization of the proteins involved in link formation obtained at 50% and 100% protein sequence identity is shown in Figure 2. At the 50% threshold the 213 proteins were clustered into 46 groups comprising at least 2 proteins *per* group. As might be expected, the number of clusters decreased to 32 at the 100% sequence identity threshold. Still, this number is surprisingly high and includes several cases (32) of genes shared at least by two plasmids of different *Acinetobacter *species.

On the basis of the above assumption, each cluster was numbered and named according to the functional assignment of the most represented proteins in the cluster (Table 3). The analysis of data reported in Table 3 revealed that:

**Table 3:** Clusters of proteins exhibiting a link at 100% and/or 50% sequence identity.

		N° of nodes in the protein cluster	
Protein cluster	Function	50%	100%
1	Transposase	13	9
2	Transposase	4	3
3	Transposase	3	2
4a	Transposase	6	3
4b			3
5	Transposase	2	2
6a	Resolvase	8	4
6b			2
7	Resolvase	2	0
8	Integrase	2	2
9	Mob	2	0
10	MobS-like	4	0
11	MerC	7	7
12	Mercuric ion transport	8	8
13a	Mercuric ion transport	8	5
13b			2
14	MerD	8	7
15	Mercuric ion reduction	8	5
16	MerE	8	8
17	MerE-like protein	8	7
18a	MerR	8	5
18b			2
19	DNA replication	11	4
20	DNA replication	3	0
21	DNA replication	2	3
22	DNA replication	4	0
23	Transcription regulator	5	3
24	TonB dependent receptor protein	4	2
25	Inner membrane protein	6	3
26	Cro-like protein	6	2
27a	Sel-1 related protein	7	4
27b			3
28	Cytotoxic transcription repressor	2	0
29	Diaminopimelate decarboxylase	7	2
30	Cation efflux system	2	0
31	Oxacillinase	3	3
32	AraC	3	0
33	Hypothetical protein	2	0
34	Hypothetical protein	2	2
35	Hypothetical protein	2	2
36	Hypothetical protein	3	0
37	Hypothetical protein	2	0
38	Hypothetical protein	8	0
39	Hypothetical protein	3	3
40	Hypothetical protein	2	0
41	Hypothetical protein	4	4
42	Hypothetical protein	3	0
43	Hypothetical protein	2	**2**
44	Hypothetical protein	2	2
45	Hypothetical protein	2	**2**
46	Hypothetical protein	2	2

Total nodes		213	133

1) a high number of protein clusters (1-8) were constituted by proteins involved in DNA transposition.

2) Two protein clusters (9-10), comprising 2 and 4 nodes at the 50% threshold, respectively, included proteins encoded by *mob *genes, that is genes involved in plasmid transfer and/or mobilization. These two clusters disappeared at the 100% threshold.

3) Eight protein clusters (11-18), including a high number of plasmids (8), comprised proteins related to mercury resistance.

4) Fourteen protein clusters (19-32) included proteins whose function could not be assigned to a single cellular process.

5) Lastly, protein clusters numbered from 33 up to 46 (mainly composed by only two nodes) include only hypothetical proteins.

Overall, the number of nodes *per *cluster decreased from the 50% to the 100% threshold. However, clusters 11-18 (*mer*-related proteins) maintained a high number of representatives (ranging from 5 to 8) also at the 100% threshold. This is responsible for the high number of connections existing between plasmids belonging to plasmid cluster 1 of Figure 1, which is (mainly) due to the sharing of genes involved in mercury resistance.

Concerning the 280 isolated proteins, a further analysis revealed that most of them (190) perform unknown functions and cannot be included in a functional category. Among the remaining 90 proteins, 46 of them are involved in a known information storage and processing and more precisely in translation and DNA replication, recombination and repair. The other functional categories are less frequent, except for ten sequences assigned to the COG database function "Energy production and conversion" (see [Additional file 3] for a deeper analysis).

The information stored in these networks was then used for the analyses presented in the next section.

### Phylogenetic profiling

**Figure 3:**
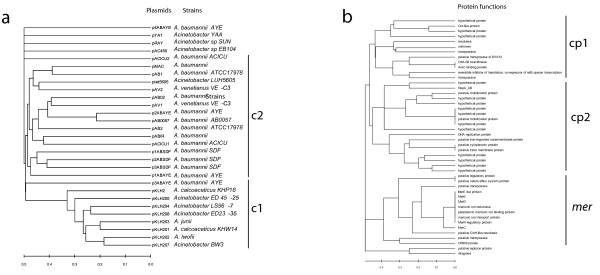
**Neighbor joining dendrograms built using the Jaccard distance matrix values between phylogenetic profiles of the proteins in the dataset (see text for details) obtained with an identity threshold of 50% for plasmids (a) and protein clusters (b)**.

In order to try to depict the relationships existing between the *Acinetobacter *plasmids, phylogenetic profiles at the 100% and 50% identity thresholds were computed. Data obtained are reported in Figure 3 and [Additional file 4].

#### Analysis of plasmid dendrograms

The analysis of the phylogenetic profiles revealed that the branching order in the plasmid dendrogram at 50% identity is in partial agreement with the subdivisions reported in the similarity network shown in Figure 1. In details, (at least) two main clusters can be identified (c1 and c2 in Figure 3a), whereby the first one (c1) embeds all the pKLH-family plasmids. This clustering is in agreement with data presented in the previous sections (Figure 1 and 2). The second cluster (c2) contains most of the plasmids belonging to *A. baumannii *strains, the two *A. venetianus *plasmids (pAV1 and pAV2) and the plasmid ptet5605 from *Acinetobacter *strain LUH5605. The remaining plasmids (those with no connection between them and with the other plasmids) and the plasmid pACICU2 and those present indifferent *Acinetobacter *strains, are not embedded in any of the two clusters.

As expected, the dendrogram built when increasing the threshold up to 100% identity possessed both longer branches and a less defined clustering of plasmids [Additional file 4]. However, in agreement with the data shown in Figure 1 and Figure 2, the pKLH plasmids formed a coherent cluster clearly separated from the other plasmids. It is quite interesting that in most cases (5 out of 6) plasmids isolated from the same strain are more related to plasmids from other strains/species rather than to the other plasmids from the same strain.

The only exception to this observation is represented by the three plasmids isolated from *A. baumannii *SDF. Indeed, all of them are embedded in the same coherent group (at 50% identity) and two of them are related also at the 100% threshold [Additional file 4].

#### Analysis of protein dendrograms

The information stored in the adjacency matrix (see Material and Methods) and previously used to build plasmids similarity dendrograms, can also be used to identify those proteins that are commonly found together (co-occurrence) in the plasmids of the dataset. The obtained protein co-occurrence dendrograms (Figure 3b and [Additional file 4]) identified three main groups at the 50% identity threshold (cp1, cp2, mer). In this dendrogram all the Mer-related proteins belong to the same cluster (*mer *cluster) comprising both proteins whose function is strictly related to mercury resistance/efflux process (their functional assignments include a cation efflux system, periplasmatic mercuric ion binding, a mercuric ion reductase and mercury ion transport) and proteins apparently not related to heavy-metal resistance and assigned with functions related either to regulation or DNA mobilization. These latter may co-occur with mercury resistance to provide accessory functions that are necessary for the transposition and the integration of the *mer *operon. In this context, it is worth noting the presence of a common *core *of *mer *genes, which is constituted by those seven proteins with distance equal to zero in the dendrogram at the 50% identity threshold (Figure 3b). The co/occurrence profile of these sequences suggests that their simultaneous presence within a plasmid might be essential for mercury resistance to occur. Indeed, this clade comprises proteins involved in mercuric ion binding, reduction and transport, although regulatory proteins are present (MerR). The main feature of cluster cp1 is the perfect co-occurrence of OXA-58 oxacillinase with a protein assigned as AraC binding protein. The former belongs to a well-known class of carbapenem-hydrolysing enzymes (OXA-type) [39], conferring reduced susceptibility to carbapenem to the bacterial host cells, whereas the latter is a regulator of transcription that changes the way in which it binds DNA when the protein forms a complex with its monosaccharide ligand, L-arabinose [40]. Quite interestingly, the finding that the genes encoding these two proteins are found always together on the same plasmid molecule might reflect the way the carbapenem resistance process is regulated and this, in turn, provide a good target for an experimental validation.

Cluster cp2 (Figure 3b) mainly includes proteins whose function has not been characterized yet and, although proposing a set of good candidates for further experimental studies, is poorly informative for the purposes of this work.

### Relationships between *Acinetobacter *plasmids and chromosomes

**Figure 4:**
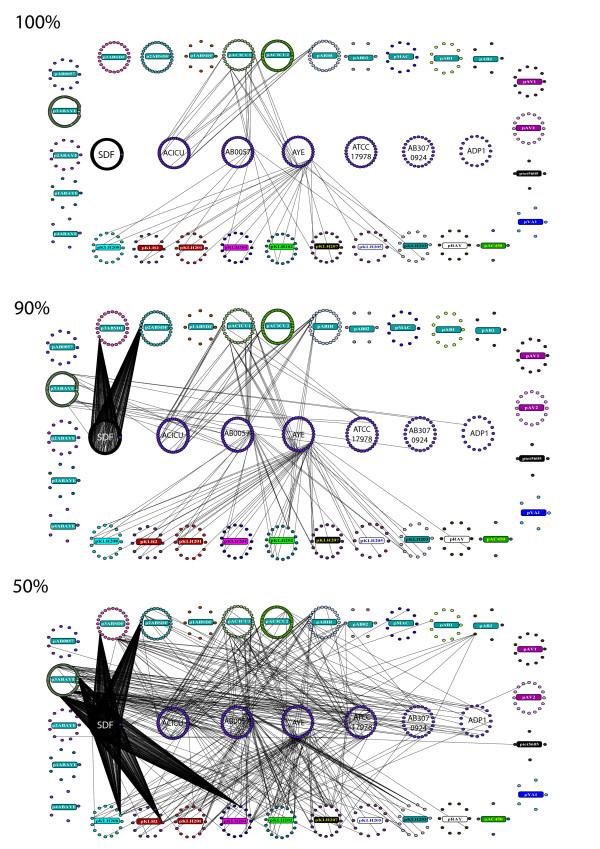
**Identity relationships between the proteins of the *Acinetobacter *plasmid and mini-chromosome proteins**. Mini-chromosomes (see text for the details of mini-chromosomes construction) are shown in the center and plasmids are circularly arranged. 100%, 90% and 50% identity threshold are shown. For clarity purposes, only the name of the corresponding strain is reported on minichromosomes. Plasmids color legend as in Figure 1.

In order to check for the existence of genes shared between plasmids and chromosomes and to look for possible indications of past and/or recent rearrangements between them, we compared the 493 plasmid proteins at different identity thresholds with all the proteins of each of the available *Acinetobacter *genomes. It is reasonable to assume that the higher the degree of sequence identity shared by a chromosomal and a plasmid encoded protein, the greater the probability that the corresponding coding gene has been exchanged between them. For this reason, similarity (identity based) networks using each of the seven *Acinetobacter *completely sequenced genome available in NCBI GenBank (*A. baumannii *17978, *A. baumannii *AYE, *A. baumannii *SDF, *A. baumannii *AB0057, *A. baumannii *AB307, *A. baumannii *ACICU and *A. baylyi *ADP1) and the 29 plasmids were constructed at different thresholds of identity. A preliminary analysis at 50% identity including all the 24,086 chromosomal encoded and the 493 plasmid encoded proteins was carried out, thereafter only those chromosomal proteins sharing at least one link with plasmid proteins were selected for comparisons at higher thresholds. In this way, we obtained a "mini-chromosome" for each of the seven *Acinetobacter *chromosomes comprising only those proteins sharing a link with (at least) one plasmid encoded protein. All the proteins (498) of these mini-chromosomes were then compared by B2N with the 493 plasmid encoded proteins. The identity networks obtained are shown in Figure 4 and [Additional file 5].

Data obtained can be summarized as follows:

1. At the lowest threshold (50%) six plasmids (pYA1, pAC450, p4ABAYE, p1ABAYE, p1ABSDF, and pAV1) did not exhibit any link with any of the chromosomally encoded proteins. Three of them (pYA1, pAC450, p4ABAYE) did not share any link also with the other plasmids (see Figure 1), suggesting that these plasmids may have originated outside these *Acinetobacter *strains. The number of isolated plasmids raised to 18 at the 100% threshold. In total, at the 50% identity threshold, 359 isolated plasmid nodes were found. Most of them (245) did not retrieve any functional assignment when probing the COG database. Most of the 114 remaining sequences (74) were found to be involved in translation and DNA replication, recombination and repair processes.

2. The pKLH-family plasmids are strongly interconnected (74 links) with the *A. baumannii *AYE chromosome, a connection degree which is maintained also at higher thresholds (90% and 100%, 42 and 20 links, respectively). Such connection, even though at a lesser extent, was also disclosed with the *A. baumanni *B0057 chromosome. More in detail, at the 100% identity threshold, connections still exist between plasmid encoded MerC, MerR and MerE and their counterparts on the *A. baumannii *AYE chromosome (in the case of *A. baumanni *B0057, only MerC is connected to its chromosomal counterpart). Lastly, two transposases from each of the two chromosomes are linked with plasmid encoded counterparts (on the pACICU1 plasmid). This finding strongly suggests a recent transfer of some genes between these, either one of the two or both chromosomes and the pKLH plasmids.

3. Concerning the DNA molecules (plasmids and chromosomes) present in the same cytoplasm, it can be highlighted that three (p1ABAYE, p2ABAYE, and p4ABAYE) of the four plasmids isolated from *A. baumannii *AYE did not share any link with the corresponding chromosome, even at the lowest threshold (50%). Hence, the corresponding genes have not been exchanged between them and the host chromosome. Their origin is unclear since they do not share any link with the other six chromosomes either. However, it is worth noting that plasmid p2ABAYE has several links with other plasmids (see Figure 1) from different *A. baumanni *strains. The fourth and larger plasmid (p3ABAYE) showed only a limited connection degree with the corresponding chromosome. Concerning the two plasmids (pAB1 and pAB2) from strain *A. baumannii *17978, pAB2 exhibited just one link with the corresponding chromosome, that disappeared at the 70% threshold (see [Additional file 4]). A similar scenario can be depicted for the three plasmids isolated from *A. baumanni *SDF. No link, neither with its corresponding chromosome nor with the six other ones, was disclosed for plasmid p1ABSDF. Each of the other two plasmids were related to the corresponding chromosome *via *a single protein (assigned as transposases), which was connected to multiple (279) almost identical proteins located on the corresponding chromosome. The only exception is represented by plasmids isolated from *A. baumanni *ACICU, which appeared to be related to their corresponding chromosome as well as to other *A. baumanni *chromosomes.

4. At the chromosome level, at the 50% threshold the most interconnected one was that of *A. baumannii *SDF (296 shared proteins), while that with least proteins shared with plasmids was the *Acinetobacter baylyi *ADP1 chromosome (4 proteins) (Table 3). At the same identity threshold (Figure 4c), also the plasmids of the pKLH-family (particularly pKLH208, pKLH2, pKLH204) and p3ABAYE showed extensive links with *A. baumanni *SDF chromosome. In this case three plasmid proteins, (GI codes 14141701 from pKLH2, 30502913 from pKLH204 and 24411190 from pKLH208), all assigned as putative transposases, were responsible for most of the links. The functional assignment of proteins linked at the threshold of 50% identity between chromosomes and plasmids (Table 4) revealed that the large majority of the proteins shared are transposases or integrase. This is mostly due to as many as 279 out of 296 proteins in *A. baumannii *SDF and 9 out of 46 in *A. baumannii *ACICU. The other proteins, excluding 26 and 59 proteins with no functional assignment and no Pfam/COG hits respectively, were assigned to possible transcriptional regulators, membrane proteins and transporters, mercury resistance and detoxification. Interestingly, *A. baumannii *AYE and *A. baumannii *AB0057 share the highest amount of *mer *related proteins and integrases (4 and 2 respectively) among all the other *Acinetobacter *genomes.

**Table 4:** Summary of all the functional categories of the proteins shared by *Acinetobacter *plasmids and chromosomes.

Functional category	*A. baumannii ATCC17978*	*A. baumannii AYE*	*A. baumannii SDF*	*A. baumannii AB0057*	*A. baumannii AB307*	*A. baumannii ACICU*	*Acinetobacter. sp. ADP1*	Total
Transposases	4	0	279	3	0	2	0	288
No Pfam hits	5	8	7	13	8	14	4	61
Uncharcterized/others	7	5	0	3	0	7	4	26
Cold-shock DNA-binding domain	2	4	1	3	4	2	3	19
Integrase	1	3	0	3	0	9	0	16
Zinc-binding dehydrogenase	1	2	1	2	2	2	2	12
Nucleoside recognition	3	1	1	1	1	1	1	9
ABC transporter	0	1	1	2	1	1	1	7
H-NS histone family	1	1	1	1	1	1	1	7
Penicillin binding protein transpeptidase domain	1	1	1	2	1	1	0	7
Mer-related protein	0	4	0	2	0	0	0	6
Haloacid dehalogenase-like hydrolase	1	1	1	1	1	1	0	6
Cation efflux family	1	1	1	1	1	1	0	6
regulatory proteins tetR-like	0	1	1	1	1	1	1	6
Catalase	1	1	0	1	1	1	0	5
Secretory lipase	1	1	0	1	1	1	0	5
AraC-like ligand binding domain	1	1	0	1	1	1	0	5
Resolvase	1	0	1	0	0	0	0	2
Proteins shared with plasmids at 50% identity	31	36	296	41	24	46	17	493

## Discussion

The genus *Acinetobacter *comprises strains and species playing an important role in different ecological niches including soil and insects, whereas particular species have emerged as opportunistic pathogens. Several *Acinetobacter *strains recovered so far harbor plasmid molecules, some of which have been correlated to the peculiar adaptation to environmental conditions (e.g., pathogenicity, resistance to heavy-metals and antibiotics, biodegradation of hydrocarbons). In this work we have analyzed the entire set of 29 available plasmid sequences (the currently available/accessible pan-plasmidome), together with seven *Acinetobacter *fully sequenced genomes, to try to depict a possible evolutionary scenario of plasmids within a bacterial genus, and to describe the horizontal gene flow within this genus.

Data obtained indicate that the 29 plasmids can be divided, by the extent of identity degree of the proteins they code for, into different groups. In particular we found that the group of pKLH-family plasmids and, to a lesser extent, those harbored by different strains of *A. baumannii*, are still interconnected at a high (90-100%) amino acid sequence identity value. This finding suggests that they might be the outcome of an evolutionary molecular history starting from ancestral plasmid backbones, which very likely underwent several and different rearrangements during the flow in different hosts, capturing and/or losing genes from their genomes. The analysis of networks constructed, excluding from the analysis those sequences responsible for heavy metal resistance and located on pKLH2-family plasmids (data not reported), reveals that pKLH2-family plasmids share a number of links higher than that exhibited with the other plasmids. Besides, some of these proteins are linked at a 100% threshold. The interconnected proteins belonging to these pKHL2-family plasmids are involved in processes, such as DNA synthesis and DNA translocation (*cinH *like), apparently not related to mercury resistance. This finding fits with the model proposed by Kholodii et al. [36] to explain the evolution of pKLH2 plasmids, according to which plasmids harboring *mer *operons are relics of an ancient plasmid that has undergone several rounds of fusions with other plasmids, followed by deletions, stabilizing the resulting mercury resistance plasmids. Hence, the occurrence of several independent recombination events might have led to the evolutionary relatedness of pHLK2, involving also the flanking regions of the *mer *operon. Moreover, these results reinforce data presented in other comparative studies and stating that plasmids from different and often geographically separated taxa may still share similar "core" genes [41-43]. Moreover, the finding that plasmid pAV2 (from *A. venetianus *VE-C3), shares some interconnections (at 100% identity) with plasmid pAB1 from a different host species (*A. baumannii*), suggests that pAV2 might be the result of recombination events that occurred between its ancestor and (at least) the ancestor of pAB1. This may be the case for several other plasmids in this study. The possibility that different plasmids may have inhabited the same host cells is emphasized by the finding that only for a few of them Inc-like proteins, causing co-existence incompatibility between plasmids, have been found (p3ABAYE, pACICU2, data not shown).

From the 50% identity threshold plasmid network, some additional information can be retrieved. In fact, pAV1 from *A. venetianus *VE-C3 shows several links with *A. baumannii *plasmids, while plasmids of the pKLH family (isolated from different *Acinetobacter *species) are linked with *A. baumannii *plasmids. Furthermore, the finding that plasmids sharing the same genes have been isolated from different strain/species may suggest the existence of both an intra- and interspecific flow of these molecules through horizontal gene transfer mechanisms. These data may suggest a time-scale of events, from the older to the most recent, paralleled by the increasing identity thresholds. In other words, some of the recombination events should have occurred very recently since the shared proteins exhibit a very high degree of sequence identity (up to 100%), whereas others (involving the genes coding for proteins sharing a low degree of sequence identity, i.e. 50%) should be more ancient.

Based on homology relationships, a total of 46 clusters were found among the proteins identified as connectors between different plasmids. At the 50% identity threshold, 8 clusters are composed by proteins involved in recombination, while the others mostly reflect the relationships between pKLH-family plasmids, being composed by genes of the *mer *operon involved in mercury resistance encoded by that plasmid family.

The finding that some plasmids (or their ancestors) might have 'inhabited' different cells belonging to different *Acinetobacter *species raises the question of what mechanism was responsible (i.e. transduction, conjugation and/or transformation) for their transfer between different hosts. Because most of the plasmids analyzed are relatively small molecules and do not harbor *tra *and/or *mob *genes, it is plausible that they might have been transmitted through transformation and/or transduction, the latter by uptake in a bacteriophage. In fact some bacteriophages, like P22 of *Salmonella typhimurium *have been shown to transduce plasmids in addition to chromosomal markers [44] other than transconjugation. Actually, the species *A. baylyi *with ADP1 (BD413) being the most widely studied strain has been shown to be naturally competent [45-50]. For other species, this property is largely unknown although in the literature there are numerous unfounded assumptions that natural competence is a general feature of the genus.

Despite the large number of links connecting most of the plasmids in our dataset, four of them, namely pAC450, pRAY, pYA1 and p4ABAYE, did not possess any of the proteins identified in the similarity network and consequently did not show any link. With the exception of pRAY, they did not share any link with the *Acinetobacter *chromosomes either. The differences in gene content exhibited by these plasmids suggests possible evolutionary pathways that did not cross those of the other *Acinetobacter *plasmids and chromosomes analyzed.

To investigate more deeply the evolutionary scenario of our plasmid dataset, we analyzed the relationships between plasmid-borne proteins and the completely sequenced genomes available. In fact, although prokaryotic plasmids have played and are still playing a key role in metabolic and genome evolution little is known about the evolutionary relationships existing between them and the chromosome(s), including the molecular rearrangements they underwent during their flow throughout the microbial community world. Data obtained in this work show the existence of extensive links between all *Acinetobacter *chromosomes and most plasmids (at 50% and even at 90% identity threshold). The finding that several connections were maintained up to the 90% identity threshold implies that the degree of divergence between the plasmid and chromosomal encoded proteins was very limited, which in turn strongly suggests that the encoding genes were exchanged (relatively) recently, independently from the possible recombination events that may have occurred between plasmids sharing the same protein coding gene. This is particularly relevant for the *A. baumanni *AYE chromosome, which seems to be the major contributor of plasmid genes, since it shares at least one link with 12 out of the 29 plasmids analyzed at a 90% identity threshold and for the *A. baumanni *SDF chromosome, which shows several links with the corresponding plasmids p2ABSDF and p3ABSDF. Even though it cannot be *a priori *completely excluded, the possibility that some of the plasmids might have inherited some genes from other species of the genus *Acinetobacter *or even of other genera, the degree of sequence similarity is sufficiently high to suggest evolutionary recent exchanges between those chromosomes and the plasmids.

It is also interesting to note that plasmids from the same host (as pAV1 and pAV2, pAB1 and pAB2, pMAC and pAB02) show links with different *Acinetobacter *chromosomes, suggesting independent evolutionary pathways not related to the particular host in which they have been isolated.

Plasmids pYA1 and pAC450, i.e. two of the three that do not share any link with the other plasmids, did not share any link with any of the chromosomes at the identity threshold of 50%, suggesting that they may have acquired/exchanged these genes from/with other bacterial chromosomes. However, the lack of knowledge on the genome sequences of their respective current hosts hampers discussion about their co-evolution with their host's chromosomes. In fact, the presence of a large pangenome for the genus *Acinetobacter *with a core genome accounting for only 50-70% of the total genome [51], strongly limits a full evolutionary reconstruction of plasmid life histories. This gap will probably be filled in the near future, when more sequence data from other representatives of this genus will be released.

In agreement with the presence of a large mobile gene pool, transposases are the most important functional category of shared proteins, especially for *A. baumannii *SDF. This result is in line with previous findings of comparative genomics [51] that showed that *A. baumannii *SDF is riddled with numerous relics of mobile elements, including transposons, insertion sequences and prophage elements. As expected, *A. baumannii *AYE shares the highest amount of *mer *related proteins and integrases among all the other *Acinetobacter *genomes. Actually *A. baumannii *AYE possesses mercury resistance genes (*mer *operon) [52], whereas all the other *Acinetobacter *strains are not apparently capable to detoxify mercury [51,53,54]. These data indicate that a HGT event might have been responsible for the appearance of resistance to heavy metal in *A. baumannii *AYE. According to this idea, this strain might have acquired the whole *mer *resistance operon and integrated in its chromosome after a recombination event (possibly with a pKLH2 plasmid). This finding is in agreement with data proposed by Osborn et al. [35] who suggested that transposition events appear to have been extensively involved in the evolution of *mer *determinants in Gram-negative bacteria. It is to be noticed that an event of HGT in the opposite direction, i.e. from the *Acinetobacter baumannii *AYE chromosome to one or more plasmids, cannot be *a priori *excluded. However, since all the other chromosomes lack the *mer *operon, we reckon the first scenario as the most parsimonious and the most probable. However, the reconstruction of the complete evolutionary scenario of the *mer *genes will be possible only when the genome sequences of strains harboring pKLH plasmids will be available.

Increasing the threshold from 90% to 100% identity resulted in the elimination of all the links between most plasmids and the chromosomes. Surprisingly, also the highly interconnected *A. baumannii *SDF chromosome lost all the links between its proteins (mostly transposases) and plasmids. This latter result might be accounted for by the hypothesis of the absence of particular structural/functional constraints acting on the transposases, leading to a (relatively) rapid diversification of proteins during evolution. On the contrary *A. baumannii *AYE, B0057 and ACICU maintained most of the links with the plasmids, revealing either strong functional constraints over the sequences of the shared proteins (mainly involved in heavy-metal resistance) or, alternatively, recent HGT events.

## Conclusions

Data obtained in this work reveal that the absence of mobilization and transfer functions in most of the *Acinetobacter *plasmids seems not to pose particular barriers to horizontal gene transfer (HGT) since they have probably a long history of rearrangements with other plasmids and with chromosomes. Furthermore, a phylogenetic profiling pipeline was applied to the whole body of plasmids encoded sequences, revealing interesting co-occurrences that, in turn, may help to shed some light in the functioning mechanisms of proteins involved in antibiotic resistance and mercury detoxification. In fact, in our opinion, this analysis provides promising candidates for further experimental validations in the field of antibiotic resistance and bioremediation. Lastly, we have shown that, by combining plasmid and chromosome similarity, identity based, network analysis, we have been able to describe an evolutionary pathway also for highly mobile genetic elements that lack extensively shared genes. In particular, we found that transposases and selective pressure for mercury resistance seem to have played a pivotal role in plasmid evolution in *Acinetobacter *genomes sequenced so far.

## Methods

### Sequence data source

The dataset used in this work is composed of all the proteins encoded by all the available completely sequenced *Acinetobacter *plasmids and chromosomes, downloaded from the NCBI ftp websites ftp://ftp.ncbi.nih.gov/refseq/release/plasmid and ftp://ftp.ncbi.nih.gov/genomes/Bacteria/, respectively (Table 1). On March 31 2009, 29 completely sequenced plasmids (whose lengths range between 2,726 and 94,413 bp) were available for a total of 493 amino acid sequences encoded. In addition, the genomic sequences of 7 *Acinetobacter *strains were also available, encoding for a total of 24,086 putative proteins.

### Network construction and phylogenetic profiling

Similarity, identity based, networks were constructed using the tools implemented in the software B2N [15]. Networks, whereby the nodes represent the proteins and the links connecting them represent the shared identity values, were visualized and analyzed using the software Visone http://visone.info/. Phylogenetic profiling dendrograms were constructed taking as input the matrix composed by all the plasmids under analysis (rows) and all the protein clusters (columns) identified [15]. Each position of the phylogenetic profile matrix will be "1" in the case a given plasmid (row) possesses (at least) one protein in the corresponding protein cluster (column), whereas it is filled with "0" in the opposite case. B2N calculates the Jaccard distance for both dimensions of the phylogenetic profiles matrix, which corresponds to the distance between plasmids in term of shared genes, and the distance between occurrence patterns of protein clusters in plasmids. The Jaccard distance matrices are then used for the construction of two neighbor-joining dendrograms. The first one describes similarities in gene content of the plasmids, the other one groups together those protein clusters with the most similar occurrence pattern within plasmids. Finally, random permutations of the original data allow to compute the statistical significance of the Jaccard distances.

### Functional Assignment

The putative functional role of unassigned proteins was automatically retrieved according to the first best hit (FBH) in a similarity search (using Blast algorithm [55]) in the COG http://www.ncbi.nlm.nih.gov/COG/ and in the PFAM database http://pfam.sanger.ac.uk/. In both cases the standalone version of the databases was used, using default parameters.

## Authors' contributions

MF, MB, AM and RF conceived the idea. MF, GB and MCP performed the analyses. All the authors participated in discussing the results and in preparing the manuscript.

## Supplementary Material

Additional file 1 **Identity relationships among all the proteins of the *Acinetobacter *plasmid dataset**. All the proteins belonging to the same plasmid (nodes) are circularly arranged and are linked to the others according to the identity value they share. Three different identity thresholds are shown (60%, 70%, 80%). Plasmids names have been colored according to the habitat of their source microorganism: yellow indicates clinical sources, green indicates environmental sources, grey indicates that habitat information was not available. Click here for file

Additional file 2 **The organization of the *mer *operon in pKLH plasmids**. Schematic representation of the organization of the *mer *operon within the pKLH plasmid family. Click here for file

Additional file 3 **Functional assignment analysis of the plasmid proteins that remained isolated during network construction**. a) COG functional assignment of the 280 proteins that remained isolated in the construction of the plasmid networks (see text for details of networks construction). In b), c) and d) the details of "cellular processes", "metabolism" and "information" categories are reported, respectively. Click here for file

Additional file 4 **Phylogenetic profiling and identification codes at 100% and 50% identity thresholds**. Neighbor joining dendrograms built using the Jaccard distance matrix values (see text for details) obtained with a threshold of 100% for plasmids (a) and protein clusters (b). Neighbor joining dendrograms of protein clusters with representative GI codes. at 50% (c) and 100% (d) identity thresholds. Click here for file

Additional file 5 **Similarity, identity based, networks of plasmid and chromosome proteins**. Similarity relationships between the proteins of the *Acinetobacter *plasmid dataset and mini-chromosome proteins (see text for mini-chromosomes dataset construction) at 60%, 70%, 80% identity thresholds. Mini-chromosomes are shown in the center and plasmids are circularly arranged. Identity thresholds are shown on the bottom right of the figure. Abbreviations: Ac. b., *Acinetobacter baumannii* Click here for file
