# Supplementary material for: Exploring the evolutionary dynamics of plasmids: the Acinetobacter pan-plasmidome
Source: BMC Evol Biol. 2010 Feb 24;10:59. doi: 10.1186/1471-2148-10-59 (PMC2848654; doi:10.1186/1471-2148-10-59)
Supplement: Additional file 1 — Identity relationships among all the proteins of the Acinetobacter plasmid dataset. All the proteins belonging to the same plasmid (nodes) are circularly arranged and are linked to the others according to the identity value they share. Three different identity thresholds are shown (60%, 70%, 80%). Plasmids names have been colored according to the habitat of their source microorganism: yellow indicates clinical sources, green indicates environmental sources, grey indicates that habitat information was not available. [file 1471-2148-10-59-S1.PDF]

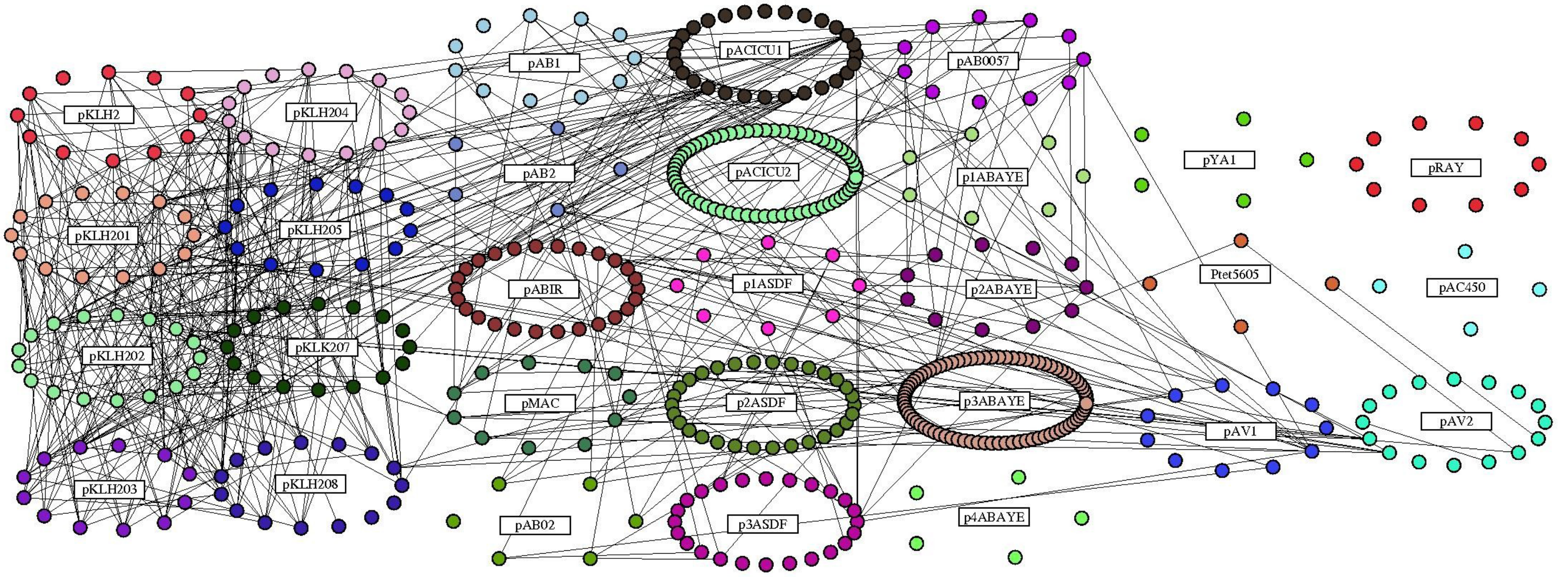

Plasmid similarity networks. The output of B2N launched on the proteins encoded by 29 plasmids of *Acinetobacter* genus. Each protein in the dataset (see Table 1) is arranged circularly with proteins from the same source plasmid; proteins from the same plasmid are shown the same colour. Identity threshold 60%

Acinetobacter baumannii	pABIR<br>pMAC<br>pAB02
Acinetobacter baumannii ACICU	pACICU1<br>pACICU2
Acinetobacter baumannii ATCC 17978	pAB1<br>pAB2
Acinetobacter baumannii AYE	p1ABAYE<br>p2ABAYE<br>p3ABAYE<br>p4ABAYE
Acinetobacter baumannii SDF	p1ABSDF<br>p2ABSDF<br>p3ABSDF
Acinetobacter baumannii AB0057	pAB0057
Acinetobacter sp. EB104	pAC450
Acinetobacter sp. SUN	pRAY
Acinetobacter venetianus	pAV1<br>pAV2
Acinetobacter LUH5605	Ptet5605
Acinetobacter BW3	pKLH207
Acinetobacter calcoaceticus KHW14	pKLH201
Acinetobacter calcoaceticusKHP18	pKLH2
Acinetobacter ED23-35	pKLH208
Acinetobacter ED45-25	pKLH205
Acinetobacter junii	pKLH203
Acinetobacter LS56-7	pKLH204
Acinetobacter lwoffii	pKLH202
*Acinetobacter YAA***	pYA1

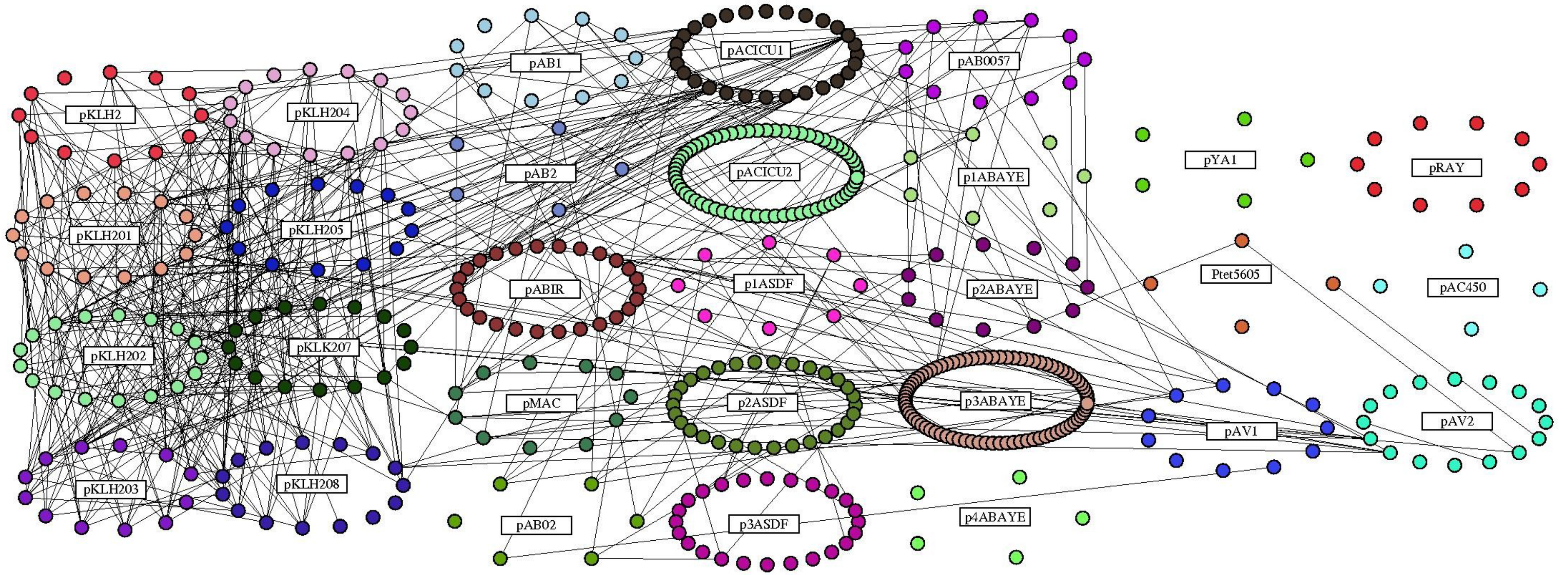

Plasmid similarity networks The output of B2N launched on the proteins encoded by 29 plasmids of *Acinetobacter* genus. Each protein in the dataset (see Table 1) is arranged circularly with proteins from the same source plasmid; proteins from the same plasmid are shown the same colour. Identity threshold 70%

Acinetobacter baumannii	pABIR<br>pMAC<br>pAB02
Acinetobacter baumannii ACICU	pACICU1<br>pACICU2
Acinetobacter baumannii ATCC 17978	pAB1<br>pAB2
Acinetobacter baumannii AYE	p1ABAYE<br>p2ABAYE<br>p3ABAYE<br>p4ABAYE
Acinetobacter baumannii SDF	p1ABSDF<br>p2ABSDF<br>p3ABSDF
Acinetobacter baumannii AB0057	pAB0057
Acinetobacter sp. EB104	pAC450
Acinetobacter sp. SUN	pRAY
Acinetobacter venetianus	pAV1<br>pAV2
Acinetobacter LUH5605	Ptet5605
Acinetobacter BW3	pKLH207
Acinetobacter calcoaceticus KHW14	pKLH201
Acinetobacter calcoaceticusKHP18	pKLH2
Acinetobacter ED23-35	pKLH208
Acinetobacter ED45-25	pKLH205
Acinetobacter junii	pKLH203
Acinetobacter LS56-7	pKLH204
Acinetobacter lwoffii	pKLH202
*Acinetobacter YAA***	pYA1

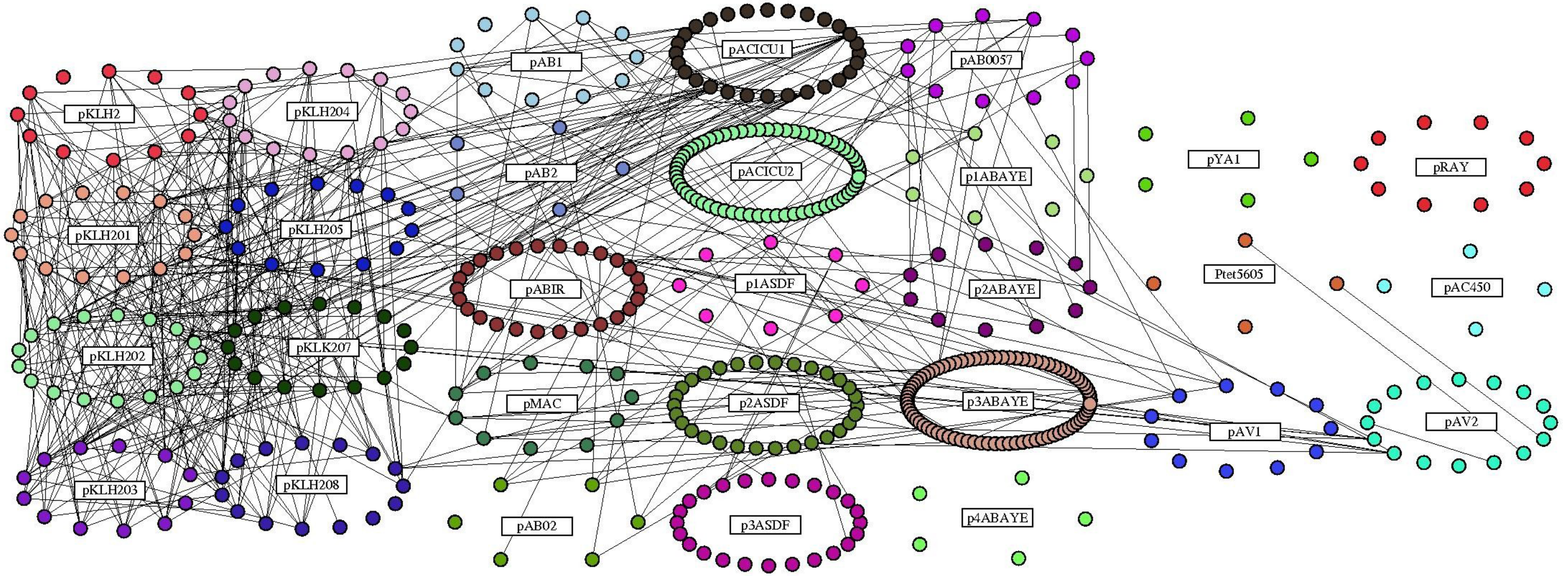

Plasmid similarity networks. The output of B2N launched on the proteins encoded by 29 plasmids of *Acinetobacter* genus. Each protein in the dataset (see Table 1) is arranged circularly with proteins from the same source plasmid; proteins from the same plasmid are shown the same colour. Identity threshold 80%.

Acinetobacter baumannii	pABIR<br>pMAC<br>pAB02
Acinetobacter baumannii ACICU	pACICU1<br>pACICU2
Acinetobacter baumannii ATCC 17978	pAB1<br>pAB2
Acinetobacter baumannii AYE	p1ABAYE<br>p2ABAYE<br>p3ABAYE<br>p4ABAYE
Acinetobacter baumannii SDF	p1ABSDF<br>p2ABSDF<br>p3ABSDF
Acinetobacter baumannii AB0057	pAB0057
Acinetobacter sp. EB104	pAC450
Acinetobacter sp. SUN	pRAY
Acinetobacter venetianus	pAV1<br>pAV2
Acinetobacter LUH5605	Ptet5605
Acinetobacter BW3	pKLH207
Acinetobacter calcoaceticus KHW14	pKLH201
Acinetobacter calcoaceticusKHP18	pKLH2
Acinetobacter ED23-35	pKLH208
Acinetobacter ED45-25	pKLH205
Acinetobacter junii	pKLH203
Acinetobacter LS56-7	pKLH204
Acinetobacter lwoffii	pKLH202
*Acinetobacter YAA***	pYA1

Color legend

A. baumannii pABIR

A. baumannii pMAC

A. baumannii pAB02

A. baumannii ACICU pACICU1

A. baumannii ACICU pACICU2

A. baumannii ATCC 17978 pAB1

A. baumannii ATCC 17978 pAB2

A. baumannii AYE p1ABAYE

A. baumannii AYE p2ABAYE

A. baumannii AYE p3ABAYE

A. baumannii AYE p4ABAYE

A. baumannii SDF p1ABSDF

A. baumannii SDF p2ABSDF

A. baumannii SDF p3ABSDF

A. baumannii AB0057 pAB0057

Acinetobacter sp. EB104 pAC450

Acinetobacter sp SUN pRAY

A. venetianus pAV1

A. venetianus pAV2

Acinetobacter LUH5605 ptet5605

A. calcoaceticus pKLH2

A.lwoffi pKLH202

Acinetobacter LS56-7 pKLH204

Acinetobacter ED23-35 pKLH208

A. calcoaceticus pKLH201

Acinetobacter BW3 pKLH207

Acinetobacter ED45-25 pKLH205

A. junii pKLH203

Acinetobacter YAA pYA1
