# Supplementary figures and images for: Exploring the evolutionary dynamics of plasmids: the Acinetobacter pan-plasmidome
Source: BMC Evol Biol. 2010 Feb 24;10:59. doi: 10.1186/1471-2148-10-59 (PMC2848654; doi:10.1186/1471-2148-10-59)

## *mer* genes

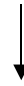

pKLH202 9471bp

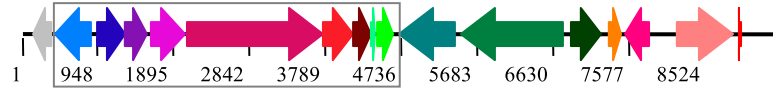

pKLH2 6838bp

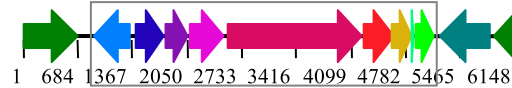

pKLH201 11191bp

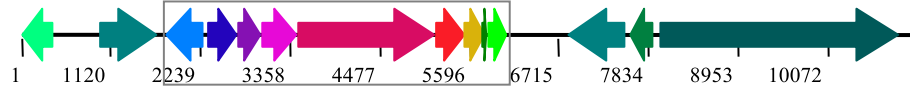

pKLH203 7195bp

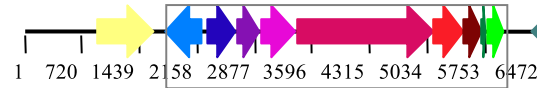

pKLH204 9489bp

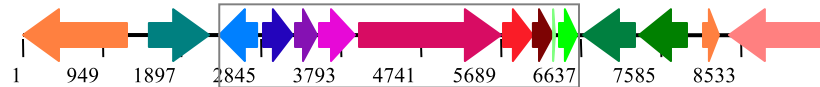

pKLH205 8561bp

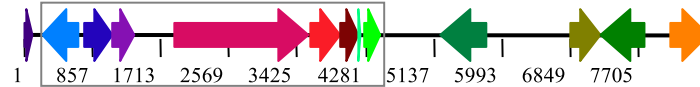

Supplement: Additional file 2 — The organization of the mer operon in pKLH plasmids. Schematic representation of the organization of the mer operon within the pKLH plasmid family. [file 1471-2148-10-59-S2.PDF]

## Slide 1
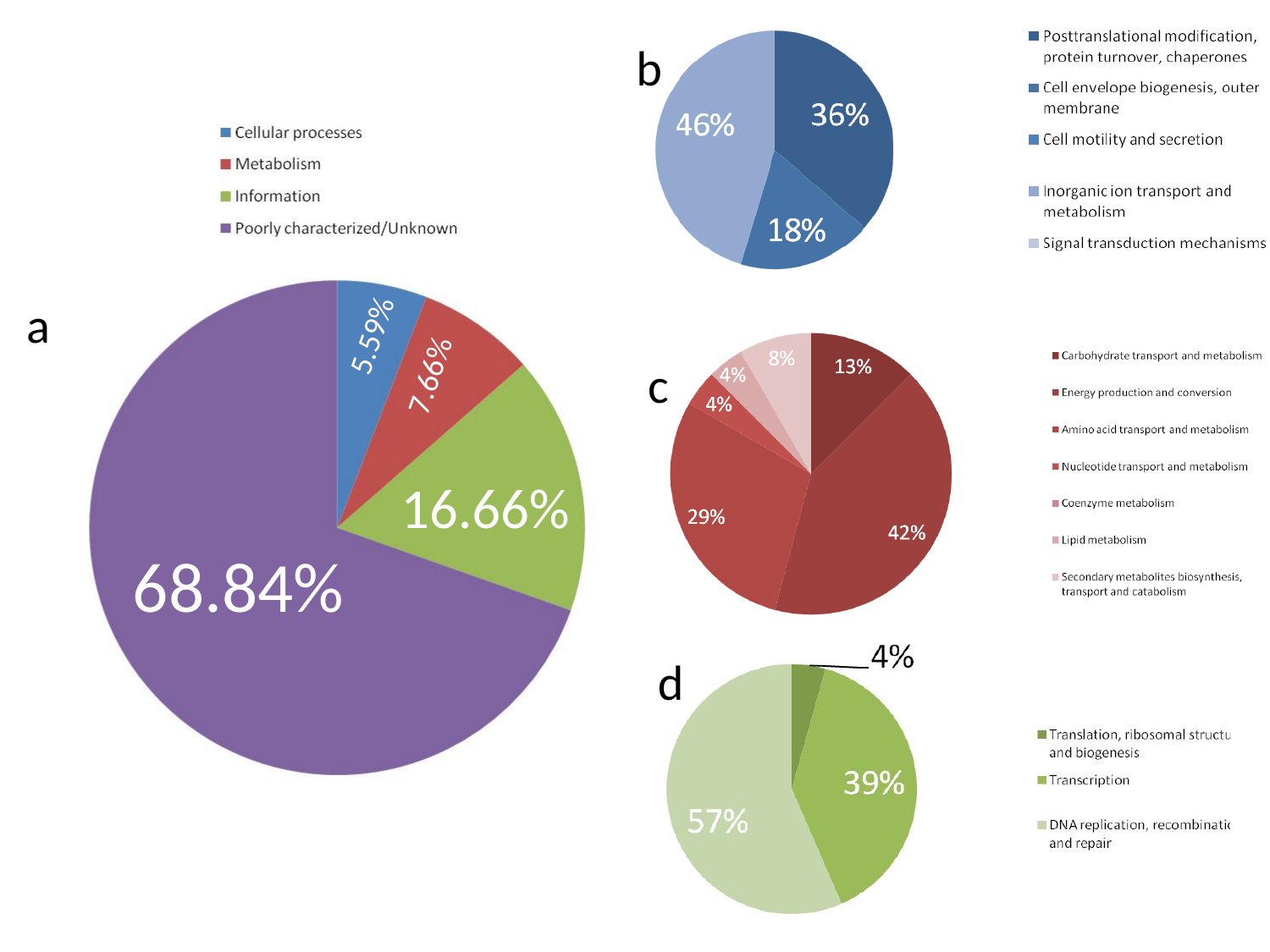

b
a
5.59%
c
7.66%
16.66%
68.84%
d

Supplement: Additional file 3 — Functional assignment analysis of the plasmid proteins that remained isolated during network construction. a) COG functional assignment of the 280 proteins that remained isolated in the construction of the plasmid networks (see text for details of networks construction). In b), c) and d) the details of "cellular processes", "metabolism" and "information" categories are reported, respectively. [file 1471-2148-10-59-S3.PPT]

(b)

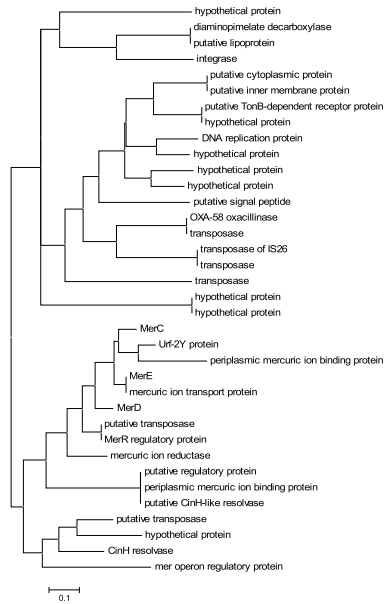

(a)

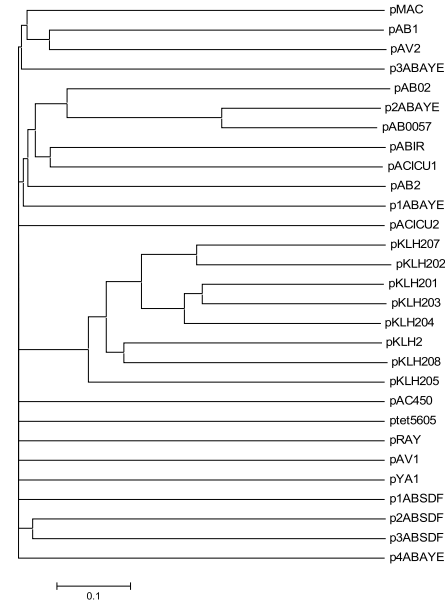

(c)

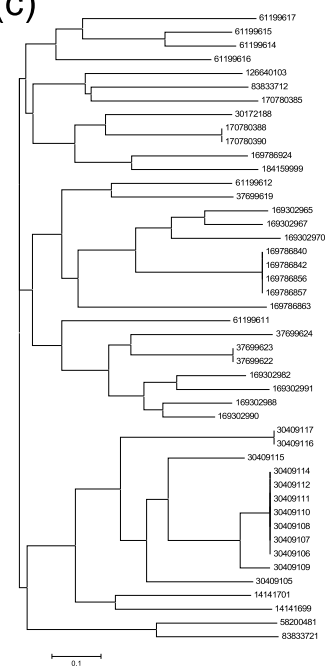

(d)

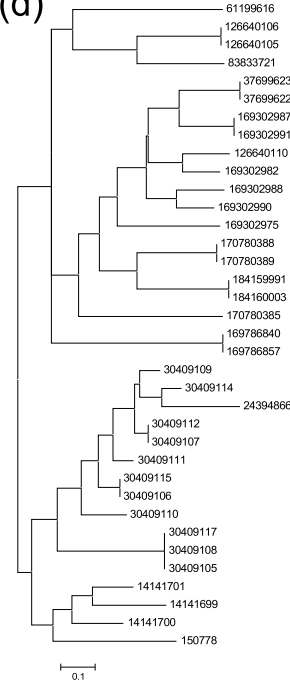

Supplement: Additional file 4 — Phylogenetic profiling and identification codes at 100% and 50% identity thresholds. Neighbor joining dendrograms built using the Jaccard distance matrix values (see text for details) obtained with a threshold of 100% for plasmids (a) and protein clusters (b). Neighbor joining dendrograms of protein clusters with representative GI codes. at 50% (c) and 100% (d) identity thresholds. [file 1471-2148-10-59-S4.PDF]

## Slide 1
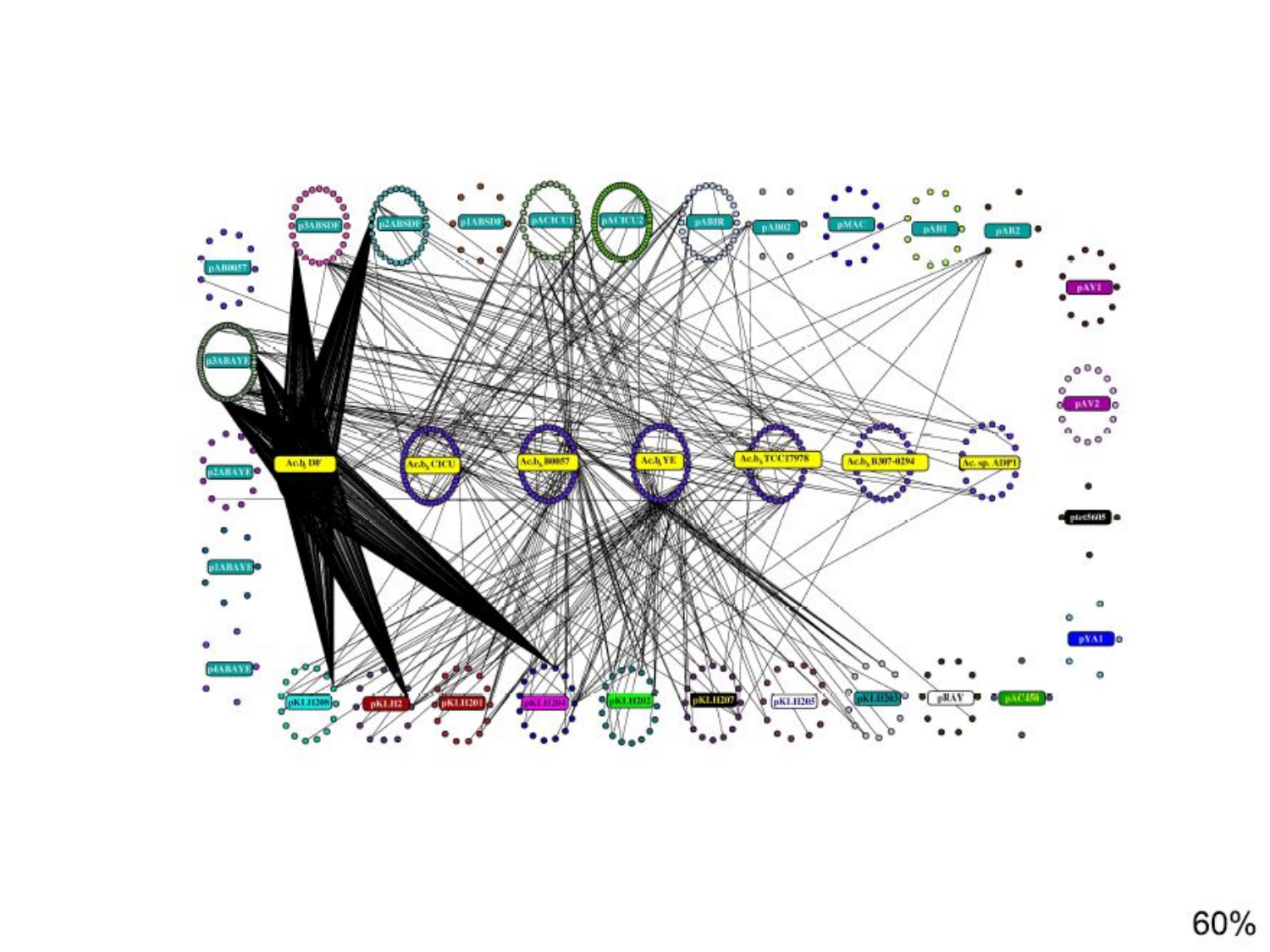

## Slide 2
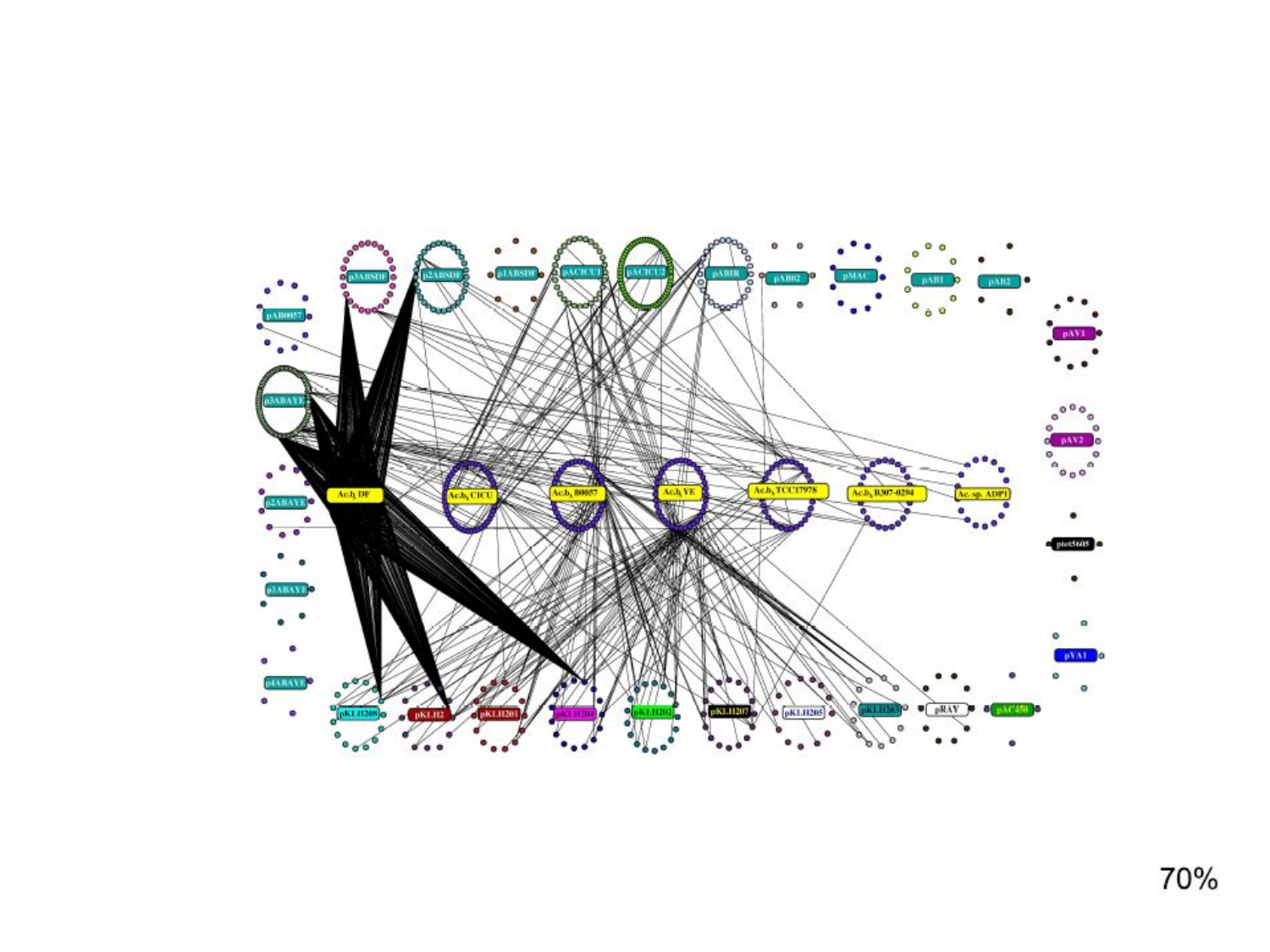

## Slide 3
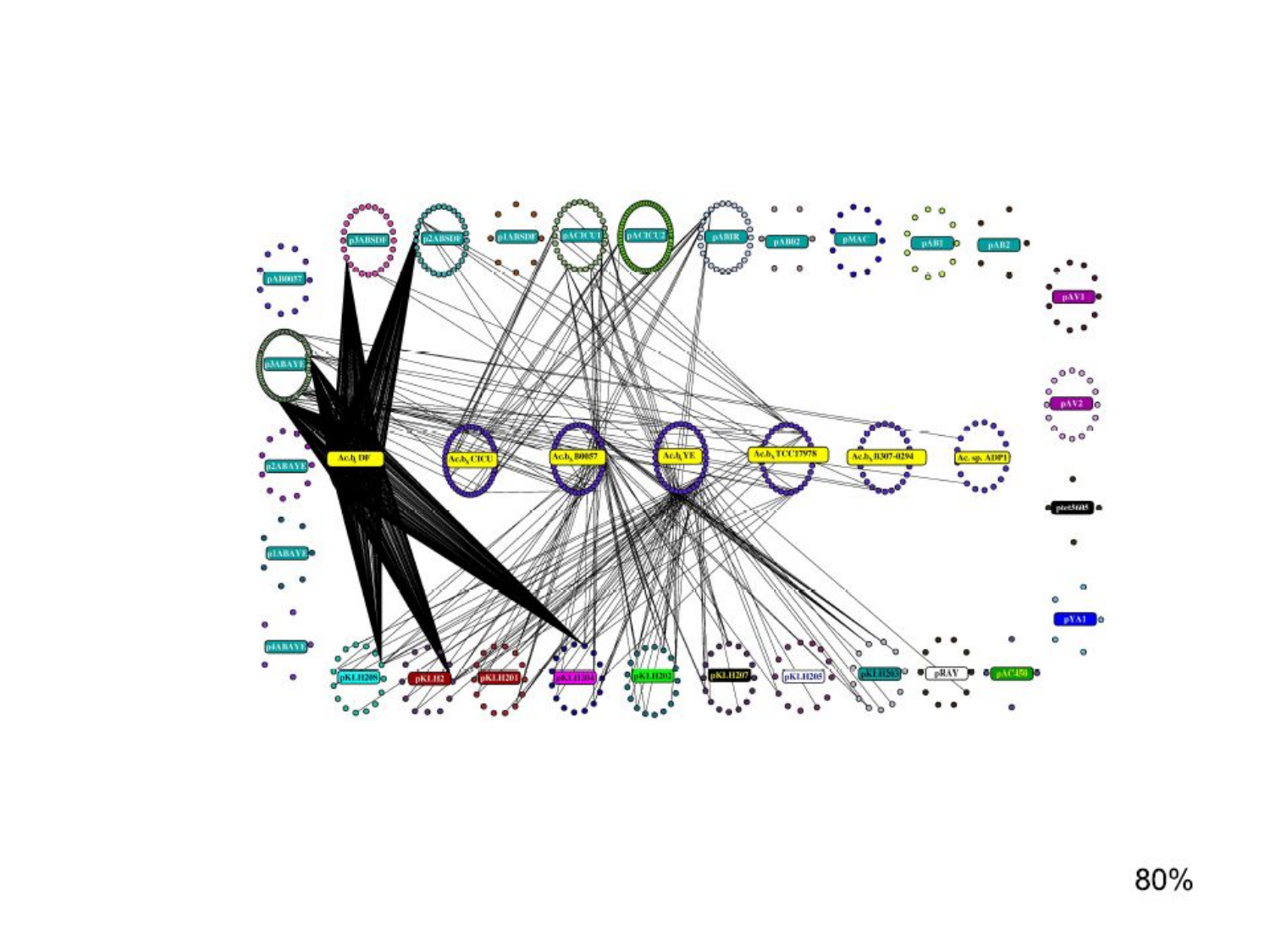

Supplement: Additional file 5 — Similarity, identity based, networks of plasmid and chromosome proteins. Similarity relationships between the proteins of the Acinetobacter plasmid dataset and mini-chromosome proteins (see text for mini-chromosomes dataset construction) at 60%, 70%, 80% identity thresholds. Mini-chromosomes are shown in the center and plasmids are circularly arranged. Identity thresholds are shown on the bottom right of the figure. Abbreviations: Ac. b., Acinetobacter baumannii [file 1471-2148-10-59-S5.PPT]
